# Mild Cognitive Impairment Identification in Geriatric and Primary Care Clinics and Driving Assessment Outcomes

**DOI:** 10.7759/cureus.118578

**Published:** 2026-10-25

**Authors:** Kourosh A Moshiri, Mahon Mahmodian

**Affiliations:** 1 Geriatrics, Eisenhower Medical Center, Rancho Mirage, USA; 2 Internal Medicine, Eisenhower Medical Center, Rancho Mirage, USA

**Keywords:** cognitive screening, driving assessment, geriatric clinic, mild cognitive impairment, older adults, primary care, quality improvement

## Abstract

Background: Mild cognitive impairment (MCI) can affect complex daily activities, including driving. This quality-improvement project compared documented MCI case counts in an outpatient geriatric clinic and a traditional primary care clinic and described Department of Motor Vehicles (DMV) referral documentation and driving assessment outcomes.

Materials and methods: A retrospective Epic electronic health record (EHR) review (Epic Systems Corporation, Verona, WI, USA) covered 2021-2023 and adults aged 65 years and older. Electronic G31.84 queries identified candidates; preserved basic and instrumental daily function and verified Montreal Cognitive Assessment (MoCA) scores of 19-25 were required for inclusion. Scanned MoCA tests and both DMV forms were located by manual chart review. Completed written and behind-the-wheel testing and overall pass/fail outcomes were distinguished. Counts and proportions were summarized descriptively, with exact binomial 95% confidence intervals (CIs) for assessment outcomes.

Results: Clinic totals were 3,233 geriatric and 3,943 primary care visits. The geriatric query identified 60 candidates. Scanned MoCA tests were located in 36; 24 without located tests were excluded. Two scores were 11, leaving 34 eligible patients. The primary care code query returned zero patients. All 34 were offered DMV assessment through shared decision making; 17 (50.0%) had documented referrals with DMV notification and initial reporting forms in their charts. The later provider form was documented for 10 of the 17. Eight completed testing (47.1% of referred patients; 80.0% of those with the later form documented). Two passed (25.0%; 95% CI, 3.2%-65.1%) and six failed (75.0%; 95% CI, 34.9%-96.8%). Eight patients continued driving without a documented referral; the reasons were undocumented.

Conclusions: Documented MCI case capture differed between clinics. Findings among assessment completers support routine clinical review of driving safety, individualized referral for formal evaluation, and coordinated follow-up. The failure proportion from this small, selected subgroup cannot be generalized to the wider MCI population. Prospective evaluation should examine patient safety, experience, and continued mobility.

## Introduction

Mild cognitive impairment (MCI) is a clinical syndrome involving cognitive decline with largely preserved independence in everyday activities. Diagnosis requires clinical and functional assessment rather than a screening score alone [1]. Driving is a complex instrumental activity of daily living, and assessment of driving concerns in cognitively impaired older adults must consider both public safety and the effects of mobility loss [2].

MCI may be associated with subtle reductions in driving performance. In an on-road study, participants with MCI performed less well than cognitively normal controls, although the differences did not represent frank driving impairment [3]. O’Connor and colleagues found that adults with MCI reported avoiding some complex driving situations, without clear differences in driving frequency or cessation across diagnostic groups [4]. These findings support individualized assessment rather than assuming that every patient with MCI is unsafe to drive. Recognition of cognitive impairment also provides opportunities for counseling and attention to modifiable dementia risk factors [5].

The purpose of this project was to compare documented MCI case identification in geriatric and primary care clinics and describe Department of Motor Vehicles (DMV) referral, assessment completion, and overall driving assessment outcomes among eligible geriatric clinic patients. The project examined documentation and follow-up among adults aged 65 years and older during 2021-2023; it did not estimate underlying MCI prevalence or comparative diagnostic accuracy.

## Materials and methods

Study design and framework

This single-center quality-improvement project used a retrospective review of electronic health records (EHRs) from 2021 to 2023 to compare documented MCI cases in an outpatient geriatric clinic and a traditional primary care clinic. Both clinics were screened for patients aged 65 years and older. The Model for Improvement described by Langley and colleagues and the Plan-Do-Study-Act (PDSA) learning cycle described by Deming informed the project framework [6,7]. This report describes the retrospective review and a proposed improvement workflow; it does not evaluate a completed intervention cycle.

Case identification and chart verification

A quality/IT staff member performed an electronic query of the Epic electronic health record (Epic Systems Corporation, Verona, WI, USA) for patients aged 65 years or older with a documented MCI diagnosis code G31.84 [8] in the geriatric and primary care clinics during 2021-2023 and supplied the extracted data to the authors. Montreal Cognitive Assessment (MoCA) documentation was identified by manually searching candidate patients' Epic charts for scanned MoCA tests [9]. Located tests were reviewed manually to verify the score and determine whether it was within 19-25, inclusive. The authors performed the manual searches and review of scanned MoCA assessments and the initial and later DMV forms in Epic. The authors also reviewed the extracted data for eligibility.

The electronic query identified 60 geriatric clinic patients with G31.84, and clinical documentation indicated intact activities of daily living (ADLs) and instrumental ADLs (IADLs) in these candidates. Final retrospective eligibility additionally required a scanned MoCA test located in Epic with a verified score of 19-25.

MoCA scores of 19-25 were selected as an operational screening interval below the conventional normal cutoff of 26 [9]. The lower bound was chosen to exclude scores of 18 or less because of concern for dementia-range impairment; MoCA developer guidance describes a commonly used boundary around 18 between MCI and Alzheimer's disease, while acknowledging score overlap [10]. Eligibility also required G31.84 and intact ADL/IADL function. This interval was not treated as a diagnostic rule: scores of 26 or higher can occur with MCI, scores of 18 or less do not independently establish dementia, and early dementia may occur within 19-25 [1,10,11].

Candidates without a located scanned MoCA were excluded because the required score criterion could not be verified retrospectively. Candidates with a located test but a score outside 19-25 were also excluded. An absent scanned document was not taken as proof that MoCA testing had never been performed. The primary care electronic search returned zero G31.84-coded patient charts, so no primary care charts proceeded to MoCA eligibility review, and none were included.

DMV referral and chart documentation

DMV assessment was offered to all 34 eligible patients. Decisions to initiate referral were made case by case through shared decision-making; the MoCA eligibility interval was not an automatic referral rule. A documented referral meant that the chart recorded a referral for DMV driving evaluation and notification using the initial standard California DMV reporting form. Seventeen patients met this definition. Both the initial California DMV reporting form and the later DMV-issued provider form were identified by manual searches of the eligible patients' Epic charts. The variable formerly labeled "DMV assessment given in clinic" meant that this initial reporting form was present in the Epic chart. The later DMV-issued form for provider completion before proceeding to assessment was a separate variable. A completed assessment meant documented completion of both written and behind-the-wheel DMV tests. Pass or fail meant the recorded overall DMV assessment outcome among completers; separate results for the two test components were not available.

Current driving status, initial reporting-form presence, later provider-form presence, completed testing, and overall assessment outcome were recorded separately. The later provider form was considered documented when it was present in Epic; form presence did not establish that it had been completed or returned. Overall assessment completion was reviewed separately. Detailed testing protocols and follow-up duration were not fully characterized in this retrospective review.

Statistical analysis

Counts and proportions were summarized descriptively. The scanned-MoCA documentation proportion used the 60 coded candidates with intact ADL/IADL function as its denominator. The referral proportion used the 34 MoCA-eligible geriatric clinic patients as its denominator; the overall completion proportion used the 17 referred patients. Completion conditional on documentation of the later DMV provider form used the 10 patients with that form documented. Pass and fail proportions used only the eight assessment completers. Two-sided exact binomial (Clopper-Pearson) 95% confidence intervals (CIs) were calculated for pass and fail proportions. No pass or fail outcome was imputed for an untested patient. No inferential comparison of clinic case counts was performed.

Improvement proposal

The review informed a proposed workflow for documenting driving concerns, coordinating appropriate DMV referrals, counseling patients and families, and tracking form documentation and completed assessments. The effectiveness of this workflow was not measured in the retrospective review.

## Results

During 2021-2023, clinic visits totaled 3,233 in geriatric care and 3,943 in primary care. The geriatric electronic query identified 60 patients with G31.84 and intact ADL/IADL function. Manual chart searches located scanned MoCA tests for 36 of these patients (60.0%); 24 (40.0%) had no scanned MoCA located and were excluded. Two of the 36 located tests had scores of 11 and were excluded, leaving 34 patients with verified scores of 19-25. The primary care query returned zero G31.84-coded patients, and zero patients were included. Table 1 summarizes visit totals and patient screening flow.

**Table 1:** Record screening and inclusion by clinic The first row reports visits; all remaining rows report patients in the candidate cohort. No G31.84-coded primary care candidates were identified. No scanned MoCA located means that the document was not found during chart review; it does not establish that testing was never performed. The 3,233 geriatric and 3,943 primary care visits represent encounters rather than unique patients. Unique clinic patient denominators and annual visit denominators were unavailable, so clinic-population prevalence was not estimated. ADL: activities of daily living; IADL: instrumental ADL; MoCA: Montreal Cognitive Assessment

Screening stage	Geriatric clinic	Primary care clinic
Total clinic visits during 2021-2023	3,233	3,943
G31.84-coded candidates with intact ADL/IADL	60	0
Candidates with a scanned MoCA located manually	36	0
Candidates excluded because no scanned MoCA was located	24	0
Candidates excluded because MoCA was outside 19-25	2 with MoCA 11	0
Patients included with verified MoCA 19-25	34	0

All 34 were offered DMV assessment. Seventeen (50.0%) had a documented referral with DMV notification and an initial reporting form in their Epic chart. Of these 17, 10 (58.8%) had the later DMV provider form documented in their charts and seven (41.2%) did not. Eight patients completed DMV assessments including both written and behind-the-wheel driving tests, corresponding to 47.1% of referred patients and 80.0% of those with the later provider form documented.

Among the eight completers, two passed the overall DMV assessment (25.0%; exact 95% CI, 3.2%-65.1%) and six failed (75.0%; 95% CI, 34.9%-96.8%). Nine referred patients had no documented completed assessment. Of the seven without the later provider form documented in their charts, two had expired licenses that were not renewed, one had voluntarily stopped driving, and four were still driving. Of the two with the later provider form documented but no completed assessment, one required form resubmission and one did not participate.

Among the 17 patients without a documented referral, nine were not driving: seven had expired licenses that were not renewed, and two had stopped voluntarily. The other eight continued driving without a documented DMV referral; the reasons for this were not documented in the EHR. Table 2 summarizes the referral and assessment stages.

**Table 2:** Department of Motor Vehicles (DMV) referral stages and driving assessment outcomes Initial form: California DMV reporting form; Later form: DMV-issued provider form; Completion required written and behind-the-wheel tests; pass/fail is the overall outcome.

Measure	Count/total	Percentage
Assessment offered	34/34	100.0%
Referral with initial DMV reporting form documented	17/34	50.0%
Later provider form documented among referred patients	10/17	58.8%
Later provider form not documented among referred patients	7/17	41.2%
Completed assessment among referred patients	8/17	47.1%
No completed assessment among referred patients	9/17	52.9%
Completed assessment with later form documented	8/10	80.0%
Passed among assessment completers	2/8	25.0%
Failed among assessment completers	6/8	75.0%

Figure 1 shows recorded driving status for the 34 eligible patients. Initial DMV reporting forms were documented for 10 of 19 patients not driving and seven of 15 patients driving. These 17 patients with documented initial forms are the same 17 referred patients in Table 2. 

**Figure 1:**
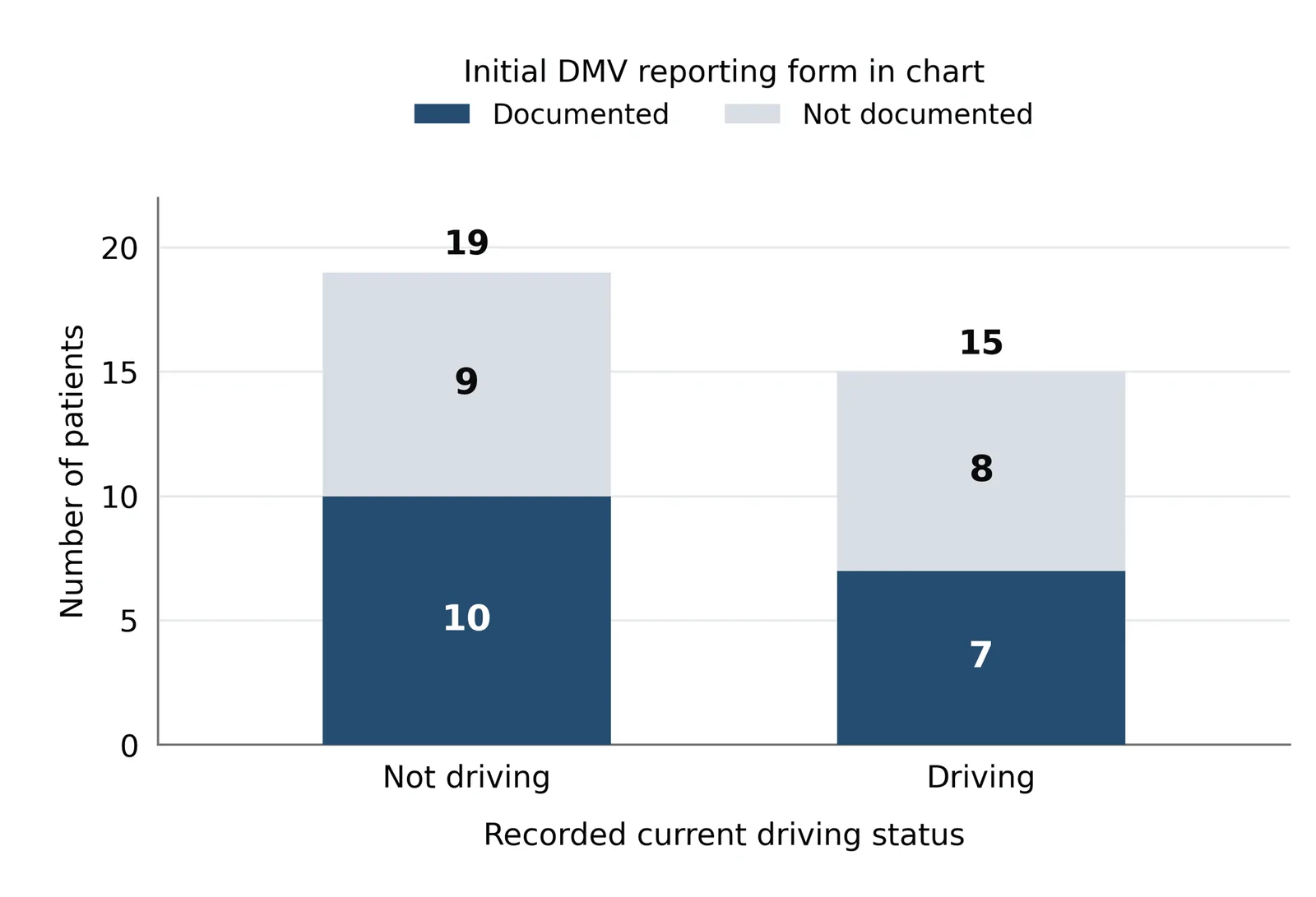
Current driving status by documentation of the initial Department of Motor Vehicles (DMV) reporting form Stacked bars report patient counts by recorded driving status and initial reporting-form documentation. Dark blue indicates documented forms and light gray indicates no documented form (total n = 34). The 17 patients with documented initial forms are the Table 2 referral cohort. Later provider forms and completed testing are separate measures.

## Discussion

Identification and documentation of cognitive impairment

The electronic search identified 60 geriatric clinic patients with G31.84 and documented intact ADL/IADL function, but scanned MoCA tests were located for only 36; 34 met the score criterion. No G31.84-coded primary care candidates were identified. These findings describe diagnosis coding and documentation-based case capture; they do not establish underlying MCI prevalence or comparative diagnostic accuracy. The 24 candidates without located scanned MoCA tests were excluded before the driving cohort was defined, so the reported referral and assessment outcomes do not describe all 60 coded patients. Documentation availability may have selected patients who differed from excluded candidates. Referral patterns, patient characteristics, availability of cognitive testing, and coding may also influence the observed clinic difference. These findings provide a local baseline for improving cognitive documentation and referral follow-up without establishing why case counts differed between clinics.

Published work supports distinguishing clinical recognition from documentation. Chodosh and colleagues found that medical records did not fully capture physicians' awareness of milder cognitive impairment and that recognition was associated with physicians' geriatrics credentials [12]. Our observed difference between clinics is consistent with an opportunity to improve assessment and documentation processes, although this review cannot determine which processes explain that difference. A geriatric approach combining cognitive testing, functional history, and information from family members or other informants may help characterize cognitive concerns more completely. Such assessment remains necessary because a MoCA score of 19-25 alone does not establish preserved functional independence or exclude dementia [1,9].

Driving assessment outcomes in the context of published evidence

The six overall DMV assessment failures among eight completers concern a selected subgroup. Driving status, form documentation, willingness to participate, and other unmeasured factors may have influenced completion. The observed failure proportion could overestimate or underestimate the proportion that would have been observed had all referred patients completed assessment. The wide 95% CI (34.9%-96.8%) reflects the small number assessed. No pass or fail outcome can be inferred for the nine referred patients without completed assessment or for the full cohort of 34 patients.

These outcomes should be interpreted alongside evidence that driving ability varies within MCI. Wadley and colleagues observed lower global and specific driving ratings in participants with MCI than in cognitively normal controls, but the differences did not amount to frank driving impairment [3]. In a study of 302 current drivers, Anstey and colleagues similarly found lower average on-road safety ratings in the MCI group, with substantial overlap between groups; driving-specific measures were more strongly associated with safety ratings than traditional neuropsychological tests [13]. Differences in patient selection, referral indications, and assessment procedures prevent direct comparison of our overall pass/fail results with those research driving ratings. Together, the evidence supports evaluation of individual driving ability rather than treating an MCI diagnosis or cognitive screening score as sufficient evidence of unsafe driving.

Driving self-report also requires careful interpretation. O'Connor and colleagues found greater avoidance of some complex driving situations among adults with MCI, without differences in driving frequency or cessation across diagnostic groups [4]. The recorded driving-status information in our review identifies patients for whom counseling and follow-up merit review. The timing of recorded driving status relative to DMV testing was not established, so Figure 1 cannot show whether testing preceded a change in driving status. A broader clinical evaluation should consider driving history, caregiver observations, vision, physical function, and medication effects alongside cognition [2].

Interpretation of incomplete follow-up

The documented completion proportion of 47.1% (8/17) describes completed driving assessments among referred patients. The initial reporting form and subsequent DMV provider form represent separate chart variables. The later provider form was documented in 10 of the 17 charts, and eight of those patients completed the assessment (80.0%). This conditional proportion describes completion among patients with the later form documented and should be reported alongside 8/17. Chart absence alone cannot establish that a form was never issued or received.

Of the seven referred patients without the later DMV provider form documented in their charts, two had unrenewed expired licenses, one had stopped driving voluntarily, and four were still driving. Among the two with the later form documented but no completed assessment, one required form resubmission and one did not participate. These observations provide information about the patients' status but do not establish every reason for noncompletion. Form presence does not establish the timing of issuance, receipt, provider completion, or submission. Incomplete EHR documentation could underascertain forms or testing outside the available records and therefore underestimate eventual assessment completion. The extent of this possible underascertainment cannot be determined from the available data.

Patients without a documented referral

Eight patients continued driving without a documented DMV referral, and the reasons were not documented in the EHR. The review, therefore, could not determine whether this reflected a decision to decline referral, clinical deferral, or another factor. This documentation gap limits interpretation of the 50.0% documented referral proportion. The assessment completion proportion of 47.1% (8/17) uses only the 17 referred patients as its denominator.

Implications for a structured referral and mobility pathway

Figure 2 presents a proposed driver diagram linking a clearer referral decision, reliable form tracking, assessment follow-up, and patient and family support to improved documentation and completion. A future PDSA pilot could test a named tracking owner, weekly review of pending forms and tests, reminders, and documented mobility counseling. Table 3 specifies referral rate, assessment completion, time to assessment, and patient/family experience measures for that pilot. These are proposed changes and measures; no prospective cycle or common follow-up window was evaluated in this retrospective review [6,7].

**Figure 2:**
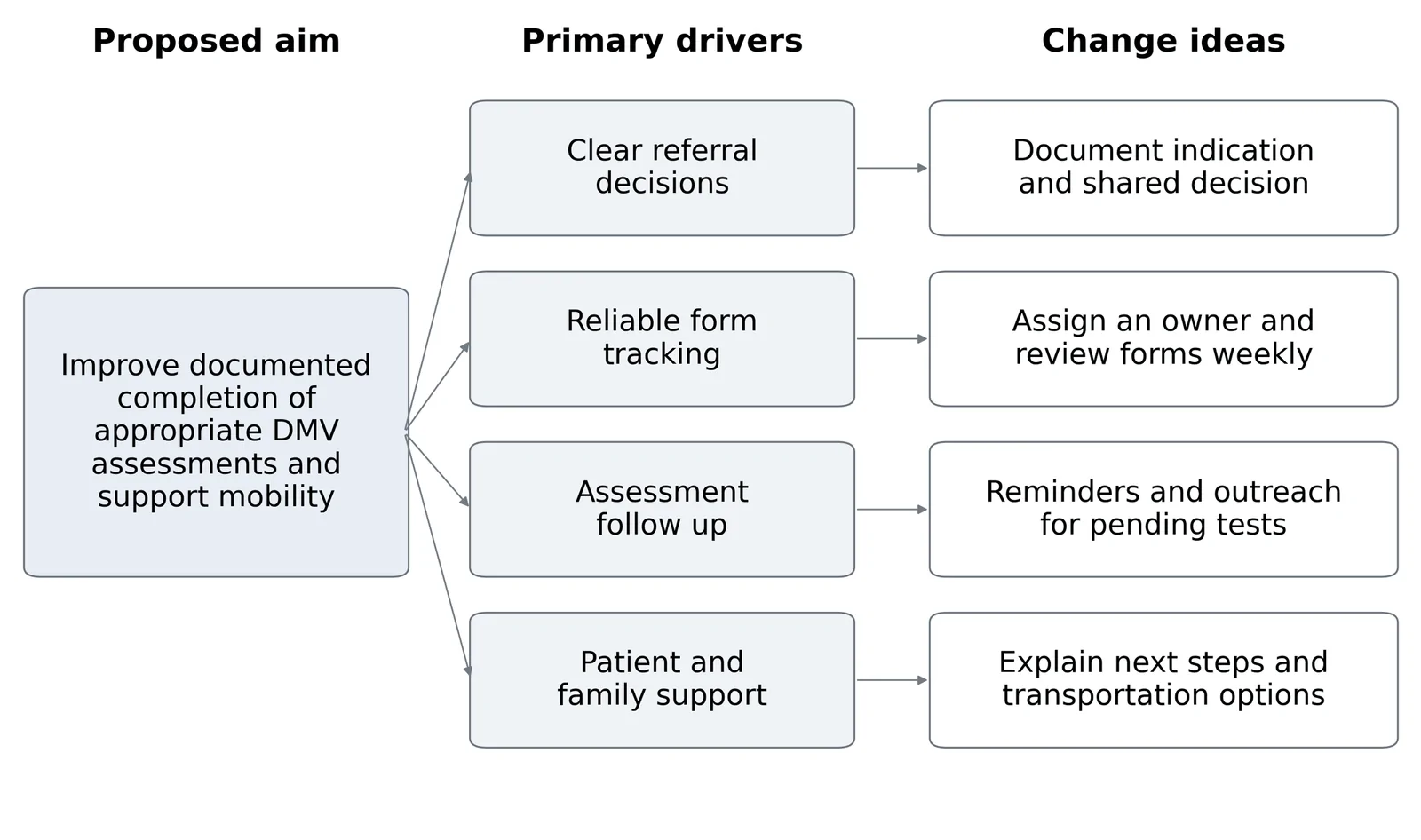
Proposed driver diagram for Department of Motor Vehicles (DMV) referral follow up and mobility support The diagram describes proposed changes for a future improvement cycle. Implementation, effectiveness, and effects on driving safety were not evaluated in the retrospective cohort.

**Table 3:** Proposed measures for a future improvement cycle All measures are proposed for prospective testing. The 90-day interval is a proposed measurement window, not an observed historical follow-up duration. A prospective baseline using the same definitions would be needed before comparing results across cycles. Referral rate is a descriptive measure and is not a goal to refer every patient regardless of clinical circumstances. DMV: Department of Motor Vehicles

Measure	Operational definition	Proposed reporting
Referral rate	Patients with a documented DMV referral divided by all patients meeting the project eligibility definition. Record current driving status and the reason for referral, nonreferral, or deferral.	Monthly counts and proportions. Historical cohort: 17/34 (50.0%).
Assessment completion	Patients with both written and behind-the-wheel tests documented within 90 days of referral divided by referred patients with 90 days of follow-up. Retain noncompleters and record their disposition.	Monthly cohorts. Historical 8/17 (47.1%) had no common 90-day window.
Time to assessment	Elapsed days from documented referral to completion of both test components among completers. Track pending referrals and their follow-up duration separately.	Median and interquartile range, with numbers completed and pending.
Patient and family experience	Brief proposed five-point ratings of understanding the referral, clarity of next steps, and adequacy of mobility planning. Record patient and caregiver responses separately.	Report each item, the response rate, and narrative concerns; collect after counseling and follow-up.

Transportation planning should accompany driving safety counseling. A systematic review by Chihuri and colleagues found that driving cessation was associated with adverse health outcomes, including increased depressive symptoms, while the observational evidence did not establish that cessation alone caused these outcomes [14]. This literature strengthens the rationale for patient and family education in our project. Clinicians can discuss transportation alternatives and access to medical appointments and social activities when driving is restricted or discontinued. A pathway should address safety and continued mobility together, with prospective evaluation of patient experience and social participation as well as referral completion.

Broader implications of timely clinical assessment

Timely recognition of cognitive concerns also creates opportunities for etiologic evaluation and counseling about modifiable dementia risk factors, as discussed in the 2024 Lancet Standing Commission report [5]. In selected patients with MCI due to Alzheimer's disease, further evaluation may identify eligibility for disease-modifying treatment. Trials of lecanemab and donanemab enrolled patients with early symptomatic Alzheimer's disease and confirmed amyloid pathology and demonstrated slowing of clinical decline in those studied populations [15,16]. These findings do not apply to all patients labeled as having MCI; treatment consideration requires appropriate diagnostic confirmation and assessment of benefits and risks. Our code-and-score case definition did not establish Alzheimer's disease etiology or treatment eligibility. These broader opportunities support careful clinical follow-up without extending the conclusions of this driving assessment review.

Limitations

This single-center retrospective quality-improvement project included a small final cohort and only eight completed driving assessments, producing wide CIs. Twenty-four of the 60 coded candidates (40.0%) were excluded because no scanned MoCA was located in Epic, and two additional candidates had scores outside the selected interval. The absence of a located scanned test does not prove that testing was never performed. Included patients may differ from excluded candidates, so selection related to test documentation limits generalizability to the full coded population. MoCA scores alone do not establish preserved functional independence or exclude dementia [1,11]. No G31.84-coded cases identified in primary care does not establish an absence of MCI in that population. Restriction to adults aged 65 years and older did not establish matched age distributions or eliminate differences in case mix. The 3,233 geriatric and 3,943 primary care visits represent encounters rather than unique patients. Unique clinic patient denominators and annual visit denominators were unavailable, so clinic-population prevalence was not estimated. The selected MoCA range may include some early dementia and exclude some patients with MCI.

Initial reporting-form documentation, later provider-form documentation, and completed testing were separate measures. Chart absence could not establish nonreceipt of a form, and form presence did not establish its completion or return. The timing of recorded driving status relative to assessment, detailed testing protocols, and follow-up duration were not fully characterized. Reasons for continued driving without a documented referral in eight patients were undocumented. Completers may not represent other patients, and the review could not determine all reasons for noncompletion or subsequent crash outcomes. No completed intervention cycle or change in crash risk was evaluated. Prospective studies with explicit eligibility criteria, consistent clinical diagnosis, defined referral procedures, and complete follow-up are needed.

## Conclusions

This review identified gaps in MCI documentation and DMV follow-up. Driving assessment outcomes highlighted potential safety concerns among patients with MCI who completed the assessment. The finding supports routine clinical review of driving safety in older adults with MCI, timely individualized referral for formal driving evaluation, and reliable follow-up of DMV forms and completed assessments. Patient and family education and transportation planning should accompany the pathway to support safety and continued mobility. The failure estimate applies to a small, selected subgroup and cannot be generalized to all older adults with MCI; crash risk and the effectiveness of universal screening were not measured. Prospective evaluation is needed to determine whether the proposed pathway improves assessment completion, patient experience, and driving outcomes.
